# Single-cell transcriptomic atlas throughout anti-BCMA CAR-T therapy in patients with multiple myeloma

**DOI:** 10.3389/fimmu.2023.1278749

**Published:** 2023-11-14

**Authors:** Yuan Xia, Qian Zhao, Xuxing Shen, Yuanyuan Jin, Jing Wang, Jianfeng Zhu, Lijuan Chen

**Affiliations:** ^1^ Department of Hematology, The First Affiliated Hospital of Nanjing Medical University, Jiangsu Province Hospital, Nanjing, China; ^2^ Department of Hematology, The Affiliated Taizhou People’s Hospital of Nanjing Medical University, Taizhou School of Clinical Medicine, Nanjing Medical University, Taizhou, China

**Keywords:** anti-BCMA CAR-T, multiple myeloma, scRNA-seq, resistance, endogenous T cell

## Abstract

**Introduction:**

The emergence of chimeric antigen receptor (CAR)-T therapy targeting B cell maturation antigen (BCMA) has improved the prognosis of patients with multiple myeloma (MM); however, the majority of patients eventually experience relapse.

**Methods:**

In this study, employing the latest single-cell RNA sequencing technology, we examined 24 bone marrow or peripheral blood samples collected throughout the course of anti-BCMA CAR-T therapy, analyzing a total of 59,725 bone marrow cells and 72,479 peripheral blood cells.

**Results:**

Our findings reveal that tumor cells in relapsed patient exhibit higher expression levels of HSP90B1 and HSPA5, and demonstrate significantly enriched pathways regarding endoplasmic reticulum stress and unfolded protein response. In the analysis of T cells, we observed that patient with impaired effector function and increased expression of immune checkpoints in endogenous T cell are more susceptible to relapse. Notably, T cells from both the bone marrow microenvironment and peripheral blood share highly similar biological characteristics.

**Discussion:**

Overall, this study provides a comprehensive atlas of endogenous immune cells, particularly in the relatively long term, after CAR-T therapy. It offers clinical evidence for a deeper understanding of the internal environment post CAR-T treatment and for identifying mechanisms underlying relapse.

## 1 Introduction

Multiple Myeloma (MM)is the second most common hematologic malignancy caused by clonal proliferation of transformed plasma cells, and typically manifested by the involvement of multiple tissues and organs (1). Even in the era of novel agents, MM remains an incurable disease. Over the past decade, chimeric antigen receptor (CAR) modified T cells, known as CAR-T cells, which could recognize and eliminate cells expressing specific antigens in an antigen-presenting cell (APC)-independent manner, has dramatically improved the survival of MM (2, 3). In a multi-center, phase 1 study in which we participated to determine the efficacy of LCAR-B38M, a CAR-T product targeting B cell maturation antigen (BCMA), in relapsed and/or refractory MM (RRMM) (Clinicaltrials.gov NO. NCT03090659, n=74), the overall response (ORR) rate reached 87.8% and the median progression-free survival (PFS) was 18 months at a median follow-up time of 47.8 months. For patients achieved partial response (PR) or better, progressive disease (PD) was subsequently observed in 43/65 (66.2%) patients, mostly during the first two years (4, 5). Similarly, our previous systematic analysis of 22 studies on anti-BCMA CAR-T therapy showed that the median PFS and overall survival (OS) were 14 months and 24 months, respectively (6). Therefore, relapse is still inevitable and remains an intractable issue following anti-BCMA CAR-T therapy.

The field of CAR-T therapy is rapidly evolving, yet the landscape of microenvironment and the underlying mechanisms of resistance has not been fully elucidated. It is demonstrated that antigenic modulation, inadequate CAR-T cell function, and immunosuppressed tumor microenvironment may contribute to the failure of CAR-T therapy (7). Of note is that, most of the investigations regarding the resistance to CAR-T therapy were performed on CAR-T cells targeting CD19, in other words, the underlying mechanisms of resistance to anti-BCMA CAR-T therapy remain largely unexplored. Due to the significant distinctions between the biology of diseases and CAR-T cells, in-depth investigation of the anti-BCMA CAR-T therapy are warranted.

Single cell RNA sequencing (scRNA-seq) enables high-throughput analysis of complex immune microenvironments by novel sequencing technologies with cell-sorting techniques (8), and have been utilized in a small number of studies regarding anti-CD19 CAR-T therapies in the last few years (9–11). In the present work, we use scRNA-seq technology to depict the landscape and temporal revolution of immune microenvironment on the matched bone marrow (BM) and peripheral blood (PB) samples, which were obtained from three MM patients underwent anti-BCMA CAR-T therapy before infusion and at 3, 6 and 9 months post infusion. We demonstrated the heterogeneity in tumor cells among different patients and identified a high-risk subpopulation that would resist CAR-T therapy. Additionally, we discovered a diminished T cell activity in relapsed patient, and clarified the significance of endogenous immunity in CAR-T therapy. These data enable us to have a more in-depth description of the single-cell transcriptomic atlas throughout the course of anti-BCMA CAR-T therapy.

## 2 Methods

### 2.1 Study population

Three patients with relapsed/refractory multiple myeloma, who had undergone at least three prior lines of therapies and received anti-BCMA CAR-T therapy in the First Affiliated Hospital of Nanjing Medical University between January 2020 and December 2020 were included in this study. Diagnosis and response assessment of MM were applied in accordance with the Revised International Myeloma Working Group (IMWG) criteria (12, 13). Exclusion criteria: Abnormal leukocyte counts or proportions before CAR-T therapy; patients with chronic infections or concurrent acute infections at each time point; patients with a history of autoimmune diseases or other types of tumors. Follow-up of all patients were conducted until January 2022.

The research protocol adhered to the principles outlined in the Declaration of Helsinki and received approval from the institutional review board of the First Affiliated Hospital of Nanjing Medical University Ethics Committee (No. 2020SR589). Informed consents were obtained from all patients before their participation in this study.

### 2.2 Single-cell suspension preparation

Bone marrow aspirate and peripheral blood were obtained before lymphodepleting chemotherapy (0 month) and at 3, 6, and 9 months after CAR-T infusion. Specimens were stored in the GEXSCOPE Tissue Preservation Solution (Singleron) and promptly dispatched to the Singleron Lab. The specimens underwent triple washing and were subsequently filtered through a 40μm-pore strainers (Falcon). After centrifugation, the supernatant was removed and the pellets were resuspended in PBS (Hyclone). Cells were incubated with red blood cell lysis bufier (Roche) to remove the erythrocytes according to the manufacturer’s instructions. The cell suspension was centrifuged and finally re-suspended with PBS. Trypan blue dye (Sigma) was used to assess the activity and density of the single-cell suspension under a microscope.

### 2.3 Sequencing library preparation and single-cell RNA sequencing

Single-cell suspensions at a concentration of 1×10^5^ cells/mL in PBS (HyClone, Shanghai, China) were prepared and loaded onto microfluidic devices. scRNA-seq libraries were constructed following the GEXSCOPE^®^ protocol by using the GEXSCOPE^®^ Single-Cell RNA Library Kit (Singleron Biotechnologies) and the Singleron Matrix^®^ Automated Single-Cell Processing System (Singleron Biotechnologies). Individual libraries were diluted to a concentration of 4 ng/µL and then pooled for sequencing. The pooled libraries were sequenced on an Illumina NovaSeq 6000 platform, generating 150 bp paired-end reads.

### 2.4 Primary analysis of scRNA-seq raw read data

The generation of gene expression profiles from raw reads was executed using CeleScope v1.5.2 (Singleron Biotechnologies) with its default parameters. Briefly, Barcodes and Unique Molecular Identifiers (UMIs) were extracted from R1 reads, followed by data correction. Subsequently, adapter sequences and poly A tails were trimmed from R2 reads. The trimmed R2 reads were then aligned to the GRCh38 (hg38) transcriptome using STAR (v2.6.1b). Following the alignment, reads that were uniquely mapped were assigned to exons using FeatureCounts (v2.0.1). Reads that were successfully assigned and shared the same cell barcode, UMI and gene were grouped together to generate the gene expression matrix for further analysis.

### 2.5 Quality control, dimension-reduction, clustering and celltype annotation

The R package Seurat (v3.1.2) was used for quality control, dimensionality reduction and clustering. For each dataset, the expression matrix underwent filtration based on the subsequent criteria: 1) cells with a gene count less than 200 or with top 2% gene count; 2) cells with top 2% UMI count; 3) cells exhibiting mitochondrial content 50%; 4) genes expressed in fewer than five cells. Detailed data quality statistics were presented in **Supplementary Table 1**.

After filtering, a total of 59,725 BM cells and 72,479 PB cells were retained for analysis, with median gene detection counts of 766 and 616 per cell, respectively, and median UMIs of 1,927 and 1,253 per cell for BM and PB, respectively. Gene expression matrix was normalized and scaled using “NormalizeData” and “ScaleData” functions. Top 2000 variable genes were selected by “FindVariableFeatures” function for Principle Component Analysis (PCA). Cells were separated into 28 clusters for BM and 26 clusters for PB by “FindClusters” function, using the top 20 principal components and resolution parameter at 1.2. Cell clusters were visualized using Uniform Manifold Approximation and Projection (UMAP) with Seurat function “RunUMAP”. The cell type identification of each cluster was determined according to the expression of canonical cell type markers for single-cell seq data, from CellMakerDB, PanglaoDB and recently published literatures.

### 2.6 Subtyping of major cell types

To obtain a high-resolution map of B cells and T cells, cells from the specific cluster were extracted and reclustered for more detailed analysis following the same procedures described above. The clustering resolution was set at 0.8. The gene sets pertinent to the signatures used for this clustering process were listed in **Supplementary Table 2**.

### 2.7 Differentially expressed genes (DEGs) analysis

To identify differentially expressed genes (DEGs), we used the Seurat “FindMarkers” function based on Wilcoxon rank-sum test with its default settings, and the genes expressed in over 10% of the cells in both of the compared groups and with an absolute log (fold change) greater than 0.25 were designated as DEGs. The adjusted *P* value was calculated by Bonferroni Correction, and *P*<0.05 was considered statistically significant.

### 2.8 Pathway enrichment analysis

For an in-depth exploration of the potential biological functions and relevant signaling pathways of each cell type, Gene Ontology (GO) and Kyoto Encyclopedia of Genes and Genomes (KEGG) enrichment were performed using the R package clusterProfiler (v4.0.2). Enriched pathways exhibiting an adjusted *P* value less than 0.05 were considered significantly enriched. Bar plots were generated for the visualization of the enrichment. The GO terms including three categories: molecular function (MF), biological process (BP), and cellular component (CC).

### 2.9 Cell-cell interaction predicted by CellPhoneDB

Cell-cell interaction (CCI) between T cell subsets and B cell subsets were predicted using established ligand-receptor pairs through CellphoneDB (v2.1.0) (14). A permutation count of 1000 was employed to compute the null distribution for the average expression of ligand-receptor pairs in randomized cell identities. Interaction pairs with a *P* value < 0.05 were considered statistically significant.

### 2.10 Pseudotime trajectory analysis

Cell differentiation trajectory of cells was reconstructed with the Monocle2 (v2.10.0) (15). For constructing the trajectory, top 2000 highly variable genes were identified by the “Find Vairable Features” function of Seurat (v3.1.2), and dimension-reduction was performed by DDRTree. The trajectory was visualized by “plot cell trajectory” function in Monocle2.

### 2.11 UCell Gene Set Scoring

Gene set scoring was carried out utilizing the R package UCell (v1.1.0) (16). UCell scores are computed via the Mann-Whitney U test by ranking query genes according to their expression levels within individual cells. The gene set used is shown in **Supplementary Table 3**. Hallmarks including unfold protein response, protein secretion, angiogenesis, endothelial-mesenchymal transition, hypoxia, reactive oxygen species, glycolysis and fatty acid metabolism were used by UCell analysis.

### 2.12 scRNA-seq based CNV detection

The package InferCNV (17) was employed to detect the copy number variation (CNV), and non-malignant cells were used as a reference to estimate the CNV of tomor cells. Genes expressed in over 20 cells were sorted based on their chromosome loci. The relative expression values were centered to 1, using 1.5 standard deviation from the residual-normalized expression values as the floor and ceiling. A slide window size of 101 genes was applied to smoothen the relative expression on each chromosome to remove the influence of gene-specific expression. Visual representation of the inferred CNV on each chromosome’s short or long arm, or full length, was achieved through heatmaps generated by the R package pheatmap.

## 3 Results

### 3.1 Single-cell atlas of bone marrow and peripheral blood from patients underwent anti-BCMA CAR-T therapy

**Figure 1:**
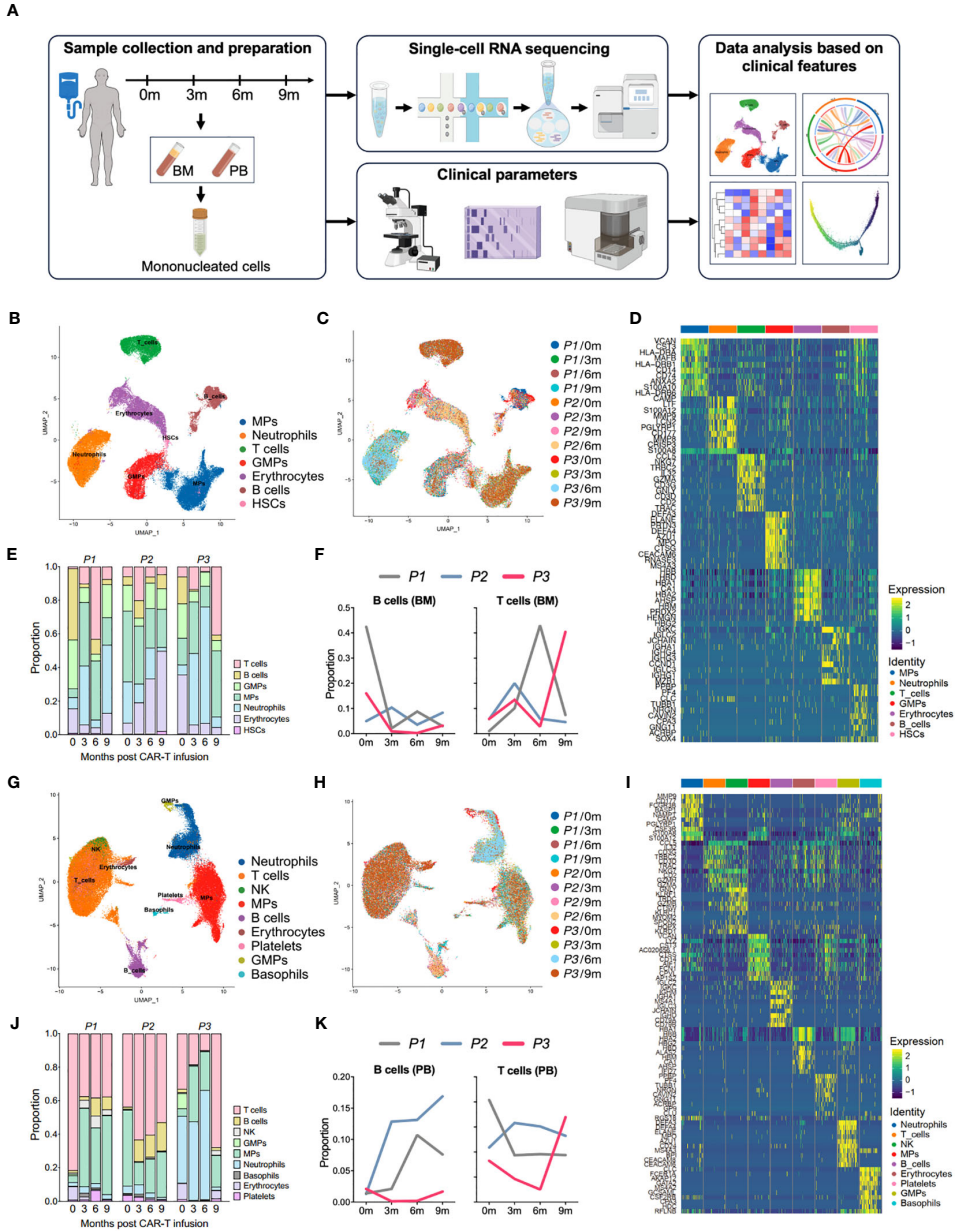
Single-cell atlas of the bone marrow and peripheral immune microenvironment throughout anti-BCMA CAR-T therapy. **(A)** Schematic overview of the experimental design of this study (created by Figdraw). **(B)** UMAP clustering of 59,725 BM cells identified 7 clusters. **(C)** UMAP plots of the BM cells colored by the sample origin. **(D)** Heatmap of differentially expressed genes of each identified cluster of the BM cells. **(E)** Bar graph showing the proportion of clusters in the BM. **(F)** Changes in proportions of B cells and T cells in the BM over time. **(G)** UMAP clustering of 72,479 PB cells identified 9 clusters. **(H)** UMAP plots of the PB cells colored by the sample origin. **(I)** Heatmap of differentially expressed genes of each identified cluster of the PB cells. **(J)** Bar graph showing the proportion of clusters in the PB. **(K)** Changes in proportions of B cells and T cells in the PB over time. BM, bone marrow; PB, peripheral blood; UMAP, uniform manifold approximation and projection.

Three patients with RRMM were treated with cyclophosphamide and fludarabine lymphodepleting chemotherapy, followed by a single infusion of autologous anti-BCMA CAR-T cells. To depicting the single-cell atlas of patients undergoing anti-BCMA CAR-T therapy, a total of 24 samples were obtained, comprising 12 bone marrow aspirates and 12 peripheral blood samples, both pre- and post-CAR-T therapy. After quality control, a total of 59,725 bone marrow cells and 72,479 PB cells were collected, and single cell RNA sequencing was conducted subsequently (**Figure 1A**). All patients achieved stringent complete response (sCR) at 3 months after CAR-T therapy, with patients 1 (P1) and 2 (P2) experiencing sustained remission within 2 years following CAR-T infusion, while patient 3 (P3) relapsed at 9 months after CAR-T treatment. Characteristics of the patients were listed in **Table 1**.

**Table 1:** Clinical characteristics of the patients.

Patient ID	P1	P2	P3
Age	67	63	51
Gender	F	M	F
Subtype	Light chain (κ)	Light chain (κ)	Light chain (λ)
Durie-Salmon stage	3A	3A	3A
ISS stage	1	2	3
R-ISS stage	1	2	2
Time since diagnosis, months	41	65	37
Prior therapy lines	3	3	4
Prior anti-CD38 mAb	No	Yes	No
Prior HSCT	No	No	No
Cytogenetic abnormalities ^a^	Negative	1q21 gain, t (11,14)	Negative
Clonal BM plasma cells, %	43.2	8.4	1.2
Clonal PB plasma cells, %	0.09	0	0
CAR^+^ cell dose, ×10^6^/kg	0.75	0.75	0.75
Grade of CRS	1	1	3
Time to relapse, months	NA	NA	9
Follow-up time, months	24	22	19

a Cytogenetic abnormalities were analyzed using fluorescence in situ hybridization (FISH) technique, and the following abnormalities were identified: 1q21 gain, del(17p), t (4,14), t (14,16), and t (11,14).

ISS, International Staging System; R-ISS, revised International Staging System; mAb, monoclonal antibody; HSCT, hematopoietic stem cell transplantation; CRS, cytokine release syndrome.

We performed dimensionality reduction by UMAP clustering analysis for the BM cells from 12 samples and sorted them into 7 cell types including B cells, T cells, mononuclear phagocytes (MPs), neutrophils, granulocyte-monocyte progenitors (GMPs), hematopoietic stem cell (HSCs) and erythrocytes. The cluster distribution was relatively consistent among different samples (**Figures 1B–D**). At 3 months after CAR-T infusion, all three patients showed a significant increase in the proportion of T cells compared to pre-infusion levels. However, by 9 months after treatment, P1 and P2 exhibited a downward trend in the proportion of T cells, while P3 showed a notable boost in T cell (**Figures 1E, F**; **Supplementary Figure 1A**).

Similarly, in the clustering analysis of PB cells from 12 samples, a total of 9 clusters were ploted, including B cells, T cells, MPs, neutrophils, NK cells, basophils, GMPs, platelets and erythrocytes, and the consistency was also observed among samples (**Figures 1G–I**). Consistently to what observed in the BM samples, analysis of PB revealed a significant increase in the proportion of T cells in P3 at 9 months after CAR-T therapy. In addition, we also noted a lower proportion of B cells in P3 in comparison to other patients. (**Figures 1J, K**). Therefore, that the altered proportion of T cells in the microenvironment may reflect the endogenous immunity following CAR-T therapy.

### 3.2 Identification of tumor cells at the single-cell level

**Figure 2:**
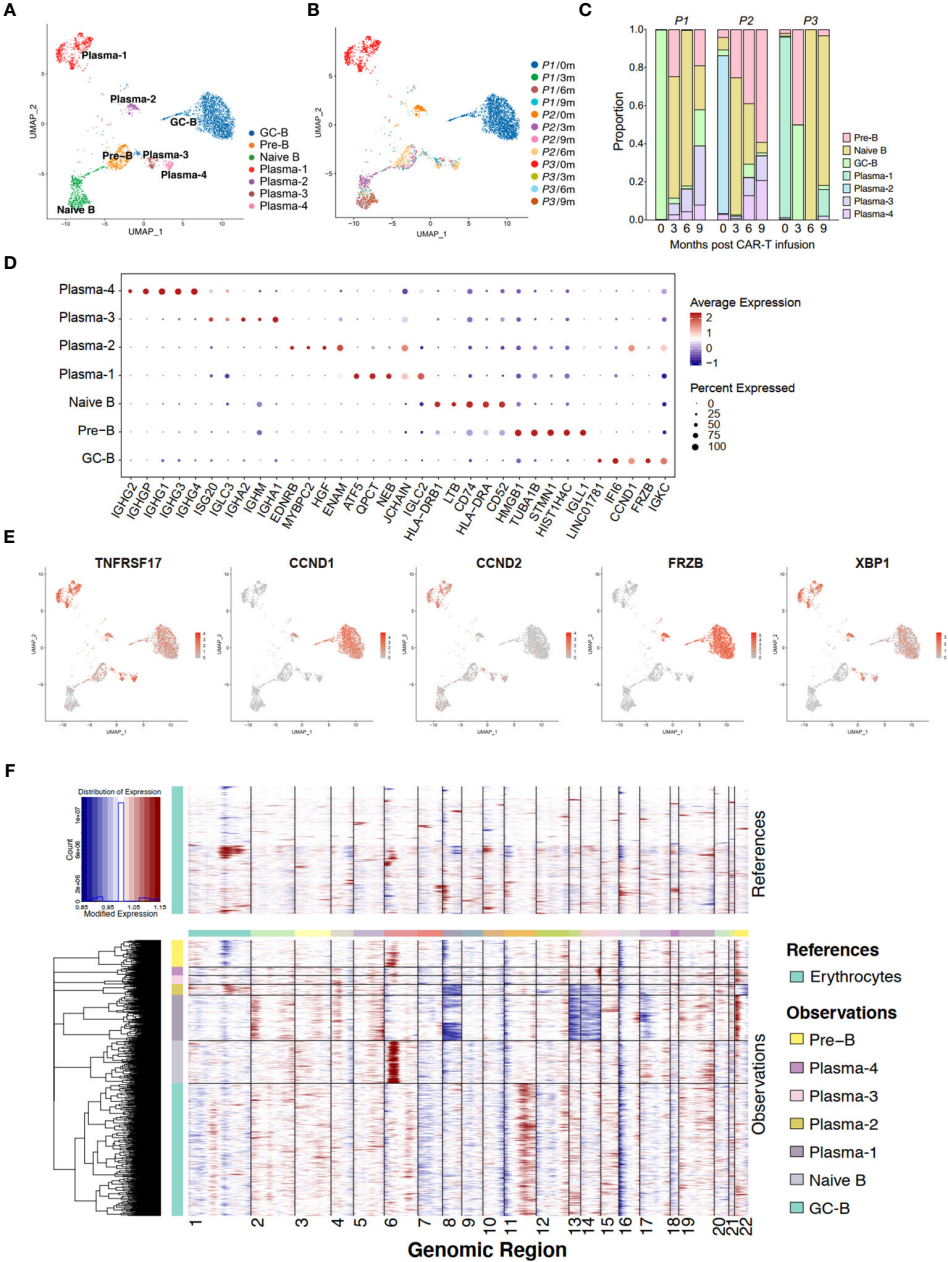
Single-cell transcriptome of bone marrow B cells in anti-BCMA CAR-T therapy. **(A)** UMAP clustering of bone marrow B cells identified 7 clusters. **(B)** UMAP plots of bone marrow B cells colored by the sample origin. **(C)** Bar graph showing the proportion of clusters in bone marrow B cells. **(D)** Dotplot of differentially expressed genes of each identified cluster of bone marrow B cells. **(E)** UMAP plot exhibiting the expression patterns of TNFRSF17, CCND1, CCND2, FRZB and XBP1. **(F)** Heatmap representing the copy number variation analysis of B cell subclusters inferred based on scRNA-seq data. UMAP, uniform manifold approximation and projection.

The tumor cells in MM are malignant transformed plasma cells, which are terminally differentiated B cells. Upon antigen activation, naive B cells undergo somatic hypermutation and class switching recombination within the germinal center, and eventually differentiate into plasma cells (18). We have further identified seven subgroups of B cells in the BM, including naive B cells, precursor B cells (Pre-B), germinal-center B cell-like cells (abbreviated as GC-B), plasma cells 1, 2, 3, and 4 (hereinafter referred to as Plasma-1 to -4) (**Figures 2A, B**; **Supplementary Figure 1B**).

Analysis of the proportions of each subgroup revealed that in the pre-CAR-T samples, sample of P1 was predominantly composed of GC-B, samples of P2 and P3 were mainly composed of Plasma-2 and Plasma-1, respectively. These three populations were found to be in absolute dominance within their respective samples (**Figure 2C**). Interestingly, we observed that the cluster GC-B were identified as tumor cells in P1, which appeared to be inconsistent with the typical characteristics of MM cells. Therefore, we analyzed the expression of several markers, including TNFRSF17 (BCMA), CCND1, CCND2, XBP1 and FRZB, that have been reported to be highly expressed in MM cells for these B cell subsets (19). It is revealed that TNFRSF17 and XBP1 were expressed in all the plasma cells and GC-B cells, and CCND1 and FRZB were mainly expressed in clusters GC-B and Plasma-2, while CCND2 was predominantly expressed in the cluster Plasma-1 (**Figures 2D, E**). Additionally, t (11, 14) (q13;q32), reported to be associated with increased expression of CCND1 (20), was detected in P2 by fluorescent *in situ* immunohybridization (FISH) assay. This was consistent with our finding that CCND1 is highly expressed in tumor cells (Plasma-2) in P2 by the scRNA-seq method.

Due to the frequent occurrence of copy number variation in MM, we conducted InferCNV analysis of different subtypes of B cells. We founded deletions on chromosomes 8,13,14, and 17 in the Plasma-1, along with amplification on chromosome 22. At the same time, we observed deletions on chromosomes 8, 13, 14, and 22 in the Plasma-2, as well as amplification on chromosome 11 in the GC-B (**Figure 2F**). These are common and distinctive chromosomal alteration observed in MM, providing further evidence of the neoplastic nature of the Plasma-1, Plasma-2 and GC-B.

### 3.3 Biology of tumor cells that resist anti-BCMA CAR-T therapy

**Figure 3:**
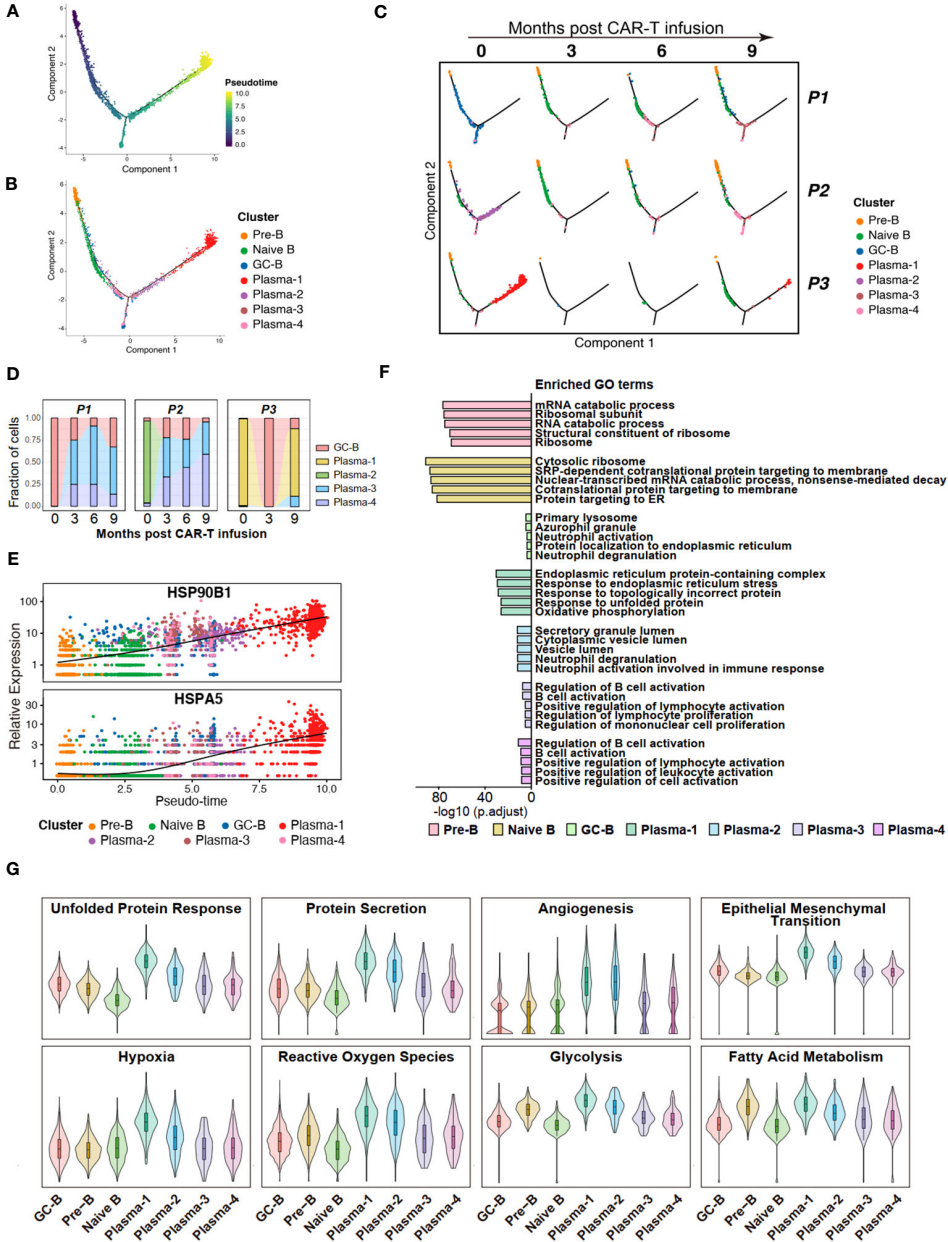
Potential features of anti-BCMA CAR-T therapy-resistant tumor cells. **(A)** Pseudotime trajectory of B cells presented according to pseudotime. Color gradient indicates pseudotime scores. **(B)** Pseudotime trajectory of B cells presented according to cell clusters. **(C)** Pseudotime trajectory of B cells for each individual sample. **(D)** Changes in proportions of tumor cells in the BM over time. **(E)** Gene expression of HSP90B1 and HSPA5 in pseudotime ordering among B cell clusters. **(F)** The top 5 terms of GO enrichment for differentially expressed genes in each B cell cluster. **(G)** UCell scores for each B cell cluster. GO, Gene ontology.

In order to elucidate the differentiation relationships among distinct B cell subpopulations, we conducted a pseudo-temporal analysis of the entire repertoire of B cell subgroups, revealing a branching differentiation pattern resembling a Y-shaped structure (**Figure 3A**). Mapping different B-cell subpopulations onto the pseudo-temporal graph reveals a progression consistent with our current understanding, wherein B cells undergo sequential differentiation from Pre-B cells to naive B cells, followed by GC-B cells, and ultimately differentiate into plasma cells (**Figure 3B**). Compared to non-neoplastic plasma cells (Plasma-3 and -4), the tumor cell populations (Plasma-1 and -2) represent a more terminal stage of differentiation (**Figures 3A, B**).

Further compositional analysis reveals that P1 and P2 exhibited a significant reduction in tumor cell count following CAR-T therapy. Conversely, in P3, the tumor cell population experienced a sharp decrease after the third month but reappeared at the ninth month, with a notably high proportion. These observations align with the clinical efficacy observed in the patients during the follow-up period, and suggest that Plasma-1 is resistant to anti-BCMA CAR-T therapy (**Figures 3C, D**). When analyzing the gene expressions across the pseudotime trajectories, we observed a notable increase in the expression of HSP90B1 and HSPA5 during B cell differentiation, with a pronounced overexpression in Plasma-1 (**Figure 3E**; **Supplementary Figure 1C**).

To uncover the characteristics and functional differences among different B cell subpopulations, we conducted GO enrichment on the DEGs of each cell subgroup (**Supplementary Table 4**) and performed UCell Gene Set Scoring analysis. The GO enrichment analysis for DEGs revealed that endoplasmic reticulum stress and unfolded protein response pathways were enriched in Plasma-1 (**Figure 3F**; **Supplementary Figures 1D, E**). Since Plasma-1 represent CAR-T therapy-resistant cells, these findings suggest a potential involvement of endoplasmic reticulum stress and unfolded protein response in CAR-T therapy resistance. According to the UCell analysis, the Plasma-1 exhibits the highest scores for unfold protein response and protein secretion, as well as for processes closely associated with tumor development, including angiogenesis, endothelial-mesenchymal transition, hypoxia, and metabolism (**Figure 3G**), implying a highly invasive nature of these cells and indicating an eventual outcome of relapse.

### 3.4 T cell characteristics hindering CAR-T therapy efficacy

**Figure 4:**
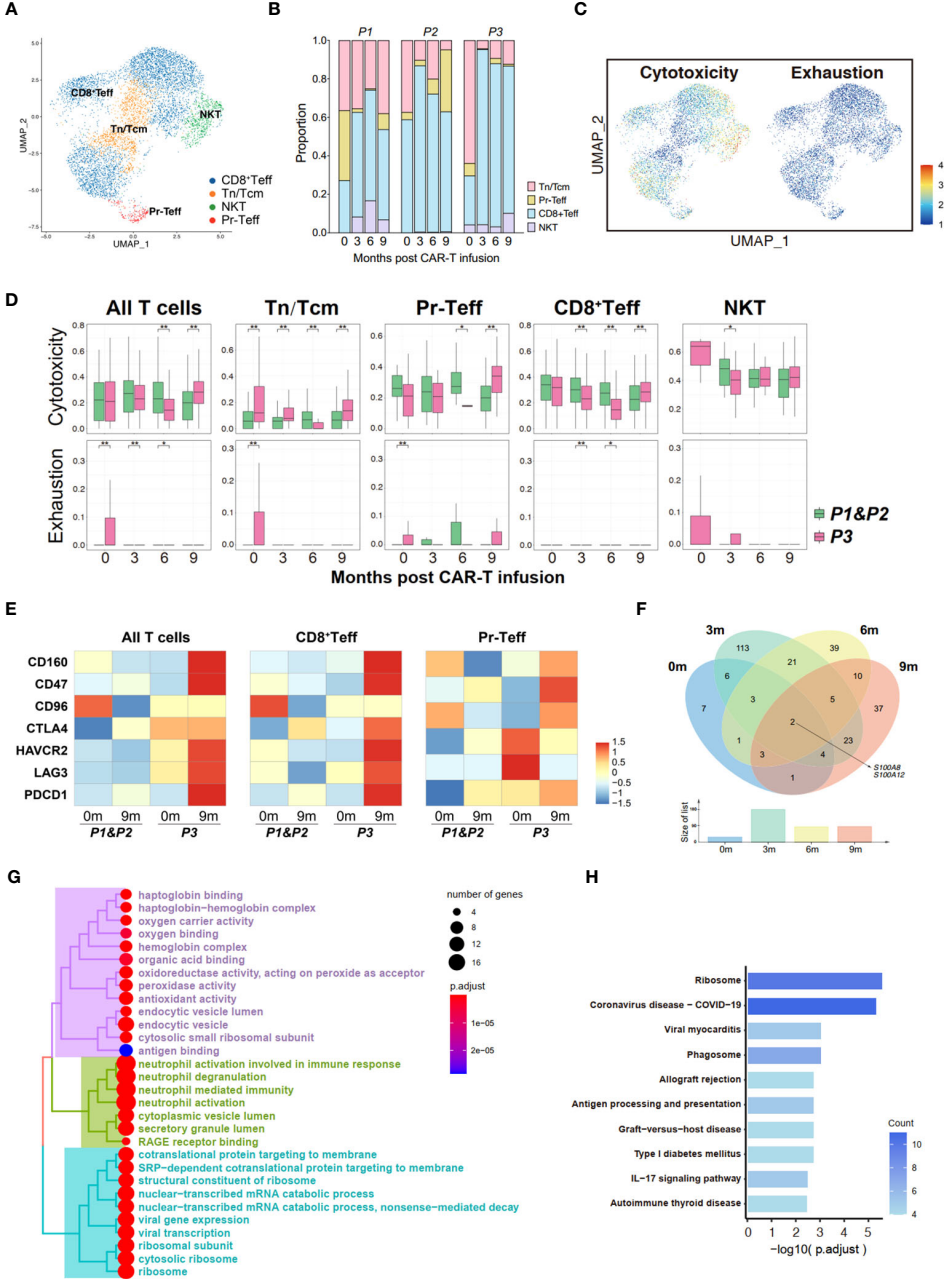
Transcriptome landscape of bone marrow T cells associated with the response to anti-BCMA CAR-T therapy. **(A)** UMAP clustering of bone marrow T cells identified 4 clusters. **(B)** Bar graph showing the proportion of clusters in bone marrow T cells. **(C)** UCell scores of cytotoxicity and exhaustion for bone marrow T cells onto the UMAP plot. **(D)** Comparisons of UCell scores for cytotoxicity and exhaustion between non-relapsed (P1&P2) and relapsed (P3) patients during anti-BCMA CAR-T therapy. **(E)** Heatmap of immune checkpoint expression in bone marrow T cells and the effector subclusters. **(F)** Venn diagram of differentially expressed genes in CD8^+^Teff at different time points following CAR-T infusion. **(G)** Treeplot of GO enrichment for differentially expressed genes in CD8^+^Teff that appear at two or more time points. **(H)** Barplot of KEGG enrichment for differentially expressed genes in CD8^+^Teff that appear at two or more time points. UMAP, uniform manifold approximation and projection; GO, gene ontology; KEGG, Kyoto Encyclopedia of Genes and Genomes. ^*^<0.05, ^**^<0.005.

In order to elucidate the changes in T cells following CAR-T therapy, a subpopulation analysis was firstly conducted on the bone marrow T cells, identifying four groups of cells: naive T cells/central memory T cells (Tn/Tcm), proliferating effector T cells (Pr-Teff), CD8^+^ effector T cells (CD8^+^Teff) and NKT cells (NKT) (**Figures 4A, B**; **Supplementary Figure 2A**). By performing UCell Gene Set Scoring analyses for T cell cytotoxicity and exhaustion throughout CAR-T cell infusion, we observed that the cytotoxicity of effector T cells (CD8^+^Teff and Pr-Teff) in P3 was lower than those of P1 and P2 during the 0-6 month period, while unexpectedly higher at 9th months. At the same time, analysis of exhaustion revealed that P3 exhibited higher scores of T cell exhaustion compared to P1 and P2. Interestingly, we also noticed that the cytotoxic score of P3 showed a downward during 0-6 months post-CAR-T infusion, and a dramatically increase at the 9th month (**Figures 4C, D**). To further figure out the immune status of bone marrow T cells, an analysis of immune checkpoint of T cells was conducted. We found that upon relapse, recurrent patient (P3) exhibited high expression of immune checkpoint molecules, including HAVCR2 (TIM3), LAG3, PDCD1 (PD-1), CD47, and CTLA4, in bone marrow T cells, especially in CD8^+^Teff (**Figure 4E**). Additionally, the expression of checkpoint molecules has shown an overall upward trend over time following CAR-T therapy in recurrent patient, which has not been observed in non-relapsed patients (**Supplementary Figure 2D**).

To illustrate the mechanisms underlying T cell dysfunction and subsequent resistance, we conducted an analysis of the differential gene expression profiles of bone marrow CD8^+^Teff in relapsed and non-relapsed patients throughout CAR-T therapy. A total of 27, 177, 84, and 85 DEGs were identified at the pre-infusion, and 3-, 6-, and 9-months post-infusion time points, respectively, in CAR-T therapy (**Supplementary Table 5**). Analysis of intersecting genes revealed that 62 genes exhibited differential expression at two time points, and 15 genes were found to be differentially expressed at three time points. Notably, two genes, S100A8 and S100A12, demonstrated differential expression across all four time points (**Figure 4F**; **Supplementary Table 6**). In addition, we also observed differential expression of another member of the S100 protein family, S100A9, at three time points throughout CAR-T therapy. Subsequently, enrichment was conducted on the DEGs that appeared two or more times. Pathways regarding neutrophil-mediated immunity, energy metabolism and ribosome were significantly enriched in GO enrichment (**Figure 4G**; **Supplementary Figure 2F**). Additionally, KEGG enrichment analysis demonstrated significant enrichment in ribosomal pathways and pathways related to immune regulation, including phagosome, antigen processing and presentation, and the IL-17 signaling pathway (**Figure 4H**; **Supplementary Figure 2E**).

### 3.5 Consistence of peripheral T cells with tumor-infiltrating T cells

**Figure 5:**
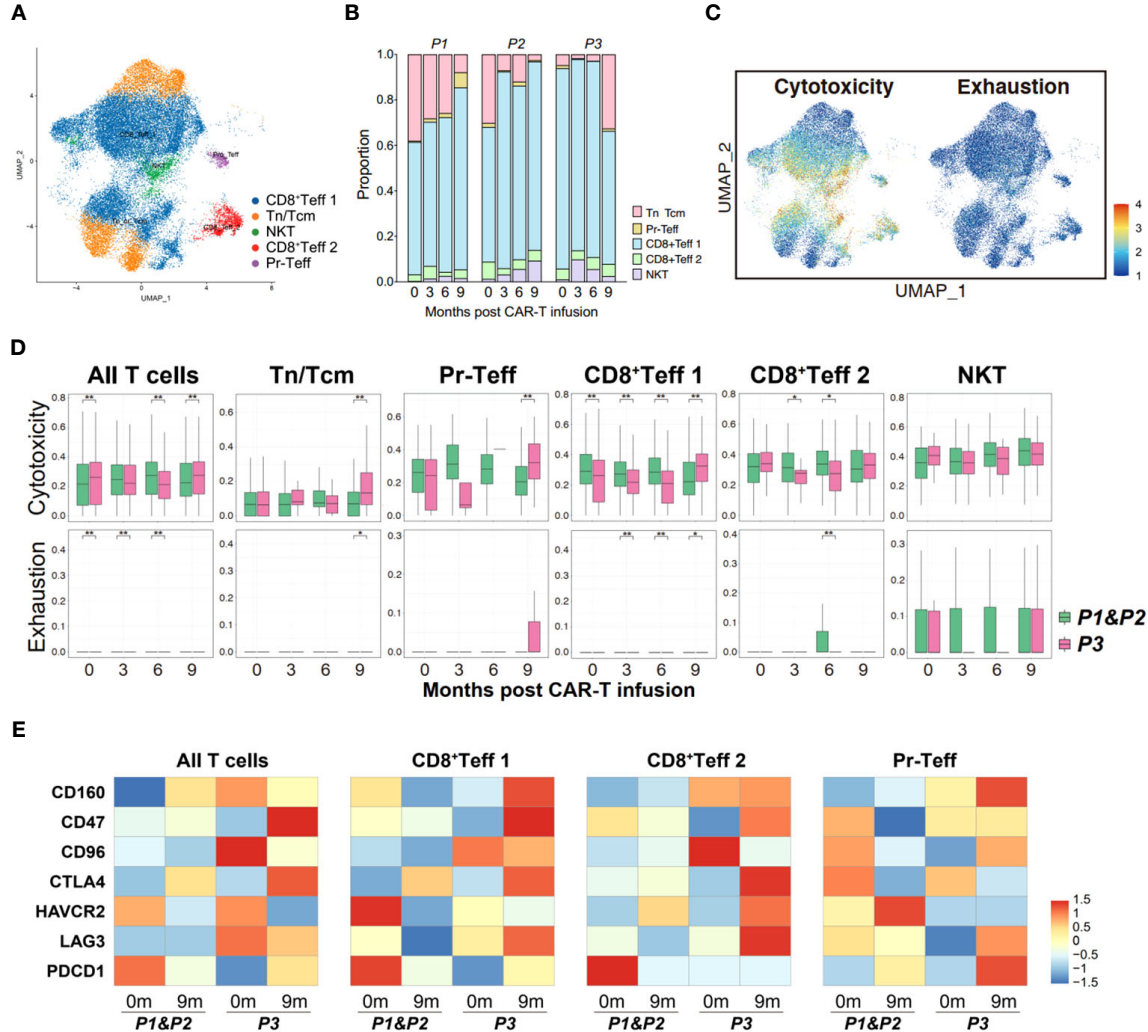
Properties of circulating T cells based on the response to anti-BCMA CAR-T therapy. **(A)** UMAP clustering of peripheral blood T cells identified 5 clusters. **(B)** Bar graph showing the proportion of clusters in peripheral blood T cells. **(C)** UCell scores of cytotoxicity and exhaustion for peripheral blood T cells onto the UMAP plot. **(D)** Comparisons of UCell scores for cytotoxicity and exhaustion between non-relapsed (P1&P2) and relapsed (P3) patients during anti-BCMA CAR-T therapy. **(E)** Heatmap of immune checkpoint expression in peripheral blood T cells and the effector subclusters. UMAP, uniform manifold approximation and projection. ^*^<0.05, ^**^<0.005.

Subsequently, we performed a similar analysis in PB cells to determine whether the immune status of T cells in circulation is consistent with those within the tumor microenvironment. Five clusters of T cells were identified in peripheral blood, including Tn/Tcm, Pr-Teff, CD8^+^Teff 1, CD8^+^Teff 2 and NKT cells (**Figures 5A, B**; **Supplementary Figure 2B**). The UCell scoring analysis revealed a high similarity in the trends of cytotoxicity and exhaustion between peripheral T cells and bone marrow T cells, suggesting consistency in the immune status of T cells between those originating from circulation and those present in the tumor microenvironment (**Figures 5C, D**). Furthermore, relapsed patient showed increased expression of immune checkpoint molecules, particularly CD47, CTLA4, LAG3, on their peripheral effector T cells during relapse (**Figure 5E**), which is similar to the expression pattern observed in bone marrow T cells. These observations suggest a strong concordance in the immune status between bone marrow and peripheral T cells.

### 3.6 Analysis of cell-cell interaction between tumor cells and T cells in CAR-T therapy

**Figure 6:**
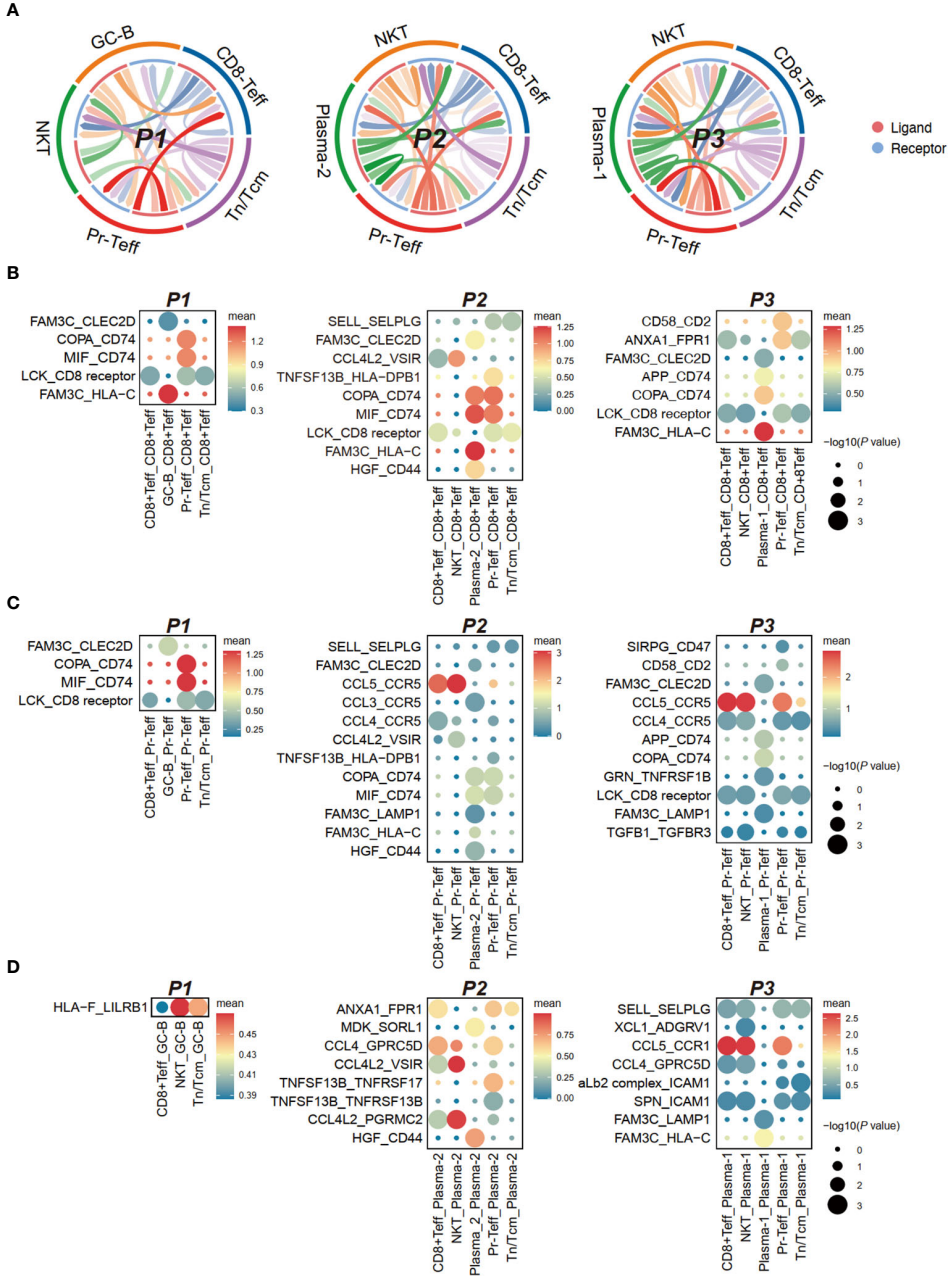
Cell communication between bone marrow T cells and tumor cells in anti-BCMA CAR-T therapy. **(A)** Chord-arrow diagram of CCI between T cells and tumor cells in different patients. **(B)** Dotplot showing the top ligand-receptor pairs based on receptors in CD8^+^Teff. **(C)** Dotplot showing the top ligand-receptor pairs based on receptors in Pr-Teff. **(D)** Dotplot showing the top ligand-receptor pairs based on receptors in tumor cells. CCI, cell-cell interaction.

CellPhoneDB in scRNA-seq enables cellular communication analysis by identifying ligand-receptor pairs and evaluating their expression to assess cell-cell interactions and signaling (14). The heterogeneity in cell-cell interaction intensity among patients undergoing CAR-T therapy was generally moderate (**Figure 6A**), prompting us to conduct a detailed analysis of ligand-receptor pairs.

Analysis of receptors on T cells revealed commonalities primarily in the form of ligand-receptor pairs involving FAM3C, including FAM3C-CLEC2D, FAM3C-HLA-C, and FAM3C-LAMP1. However, differences were observed in interactions involving CD74 in T cell subgroups. The COPA-CD74 and MIF-CD74 pairs were diminished in CD8^+^Teff and Pr-Teff cells in P3, while a significant interaction of APP-CD74 was observed, which was not observed in other patients. MIF-CD74 was found to be a significant interaction pair in both CD8^+^Teff and Pr-Teff cells of P1 and P2 (**Figures 6B, C**; **Supplementary Figures 3A, B**). Aditionally, robust interactions of SIRPG-CD47 and TGFB1-TGFBR3 were observed in P3, both of which are involved in transmitting inhibitory signals (**Figure 6C**). The receptor CCR1 on tumor cells from P3 (Plasma-1) exhibited a strong interaction with the ligand CCL5 on T cells, resulting in robust interactions between Plasma-1 and both CD8^+^Teff and Pr-Teff, which were not observed in other patients (**Figure 6D**; **Supplementary Figure 3C**).

### 3.7 Differences in quantity and function of neutrophils, MPs, and NK cells among patients with different outcomes

We witnessed a substantial rise in the proportion of neutrophils in both BM and PB of relapsed patient following CAR-T therapy, but their proportion had become similar to that of other patients upon relapse (**Supplementary Figure 4A**). Functional enrichment analysis of DEGs in neutrophils among patients with distinct efficacy revealed significant enrichment in pathways related to T cell activation, cytokine secretion and receptor activity, MHC-I receptor activity, B cell receptor signaling (**Supplementary Figures 4B, C**). Quantitative analysis of MPs in BM and PB did not seem to show significant differences (**Supplementary Figure 4A**), while enrichment analysis also identified significant enrichment in pathways involving T cell activation, B cell receptor signaling, and MHC-I receptor activity (**Supplementary Figure 4B, C**).

In addition to the two myeloid cell types mentioned above, we also conducted quantitative and functional analyses of peripheral NK cells (no distinct NK cell subpopulations were annotated in the BM) (**Supplementary Figures 5A–C**). It is worth noting that functional enrichment analysis of DEGs in peripheral NK cells between relapsed and non-relapsed patients also revealed significant enrichment in pathways such as T cell receptor binding, lymphocyte activation. Additionally, pathways related to antigen processing and presentation, phagocytosis, were prominently enriched.

## 4 Discussion

The past decade has witnessed the booming field of CAR-T therapy and the remarkable clinical activity of anti-BCMA therapy in MM, and two CAR-T products against BCMA were thereby approved by the US Food and Drug Administration (FDA) for RRMM, with a great deal of CAR-T products under development in pre-clinical and clinical trials. Despite the remarkable response rates of anti-BCMA CAR-T therapy in RRMM, durable responses remain elusive for most patients (2).

Investigations have been carried out to unveil the underlying mechanism responsible for the resistance to anti-BCMA CAR-T cells. Samur et al. (21) performed scRNA-seq for one patient who relapsed after initial anti-BCMA CAR-T therapy and identified a CAR-T-resistant clone with biallelic loss of BCMA resulting from deletion of one allele and mutation of another. In the KarMMa study, loss of BCMA on tumor cells was observed in 3/71 (4%) of the patients at progression by immunohistochemistry, while the vast majority suffered BCMA-positive relapse, suggesting BCMA antigen loss as an infrequent cause of escape from anti-BCMA CAR-T therapy (22). By contrast, antigen loss was more frequent in anti-CD19 CAR-T therapies at relapse, accounting for nearly half the patients (7), indicating that the mechanisms underlying relapse following anti-BCMA CAR-T therapy may be different from the findings we have currently observed in anti-CD19 CAR-T therapy. Moreover, high risk cytogenetics such as del(17p) or gain(1q21) were reported to be associated with reduced response to anti-BCMA CAR-T therapy (23), whereas in our study, relapsed patient was devoid of these high-risk cytogenetic abnormalities, suggesting the complexity of the mechanisms predisposing the resistance against this remedy. Herein, we applied scRNA-seq on paired bone marrow and peripheral blood cells from patients pre- and post-CAR-T therapy targeting BCMA, in the hope of a more detailed description of microenvironment in CAR-T-treated MM.

In the present work, we identified a MM subpopulation characterized by activated endoplasmic reticulum stress and upregulation of HSP90B1 and HSPA5, which confers resistance to anti-BCMA CAR-T therapy. Misfolded or unfolded proteins can accumulate in the endoplasmic reticulum (ER), triggering ER stress. To counteract this, the ubiquitin-proteasome system is activated to identify and degrade these proteins, thereby eliminating the accumulation of non-functional proteins within the cell. Excess production of abnormal immunoglobulins by MM cells places them in an ER stress state, therefore activating the unfolded protein response (UPR) to reduce the accumulation of misfolded or unfolded proteins in the ER and restore ER homeostasis (24). HSP90B1, also referred to as glucose‐regulated protein 94 (GRP94), is a molecular chaperone that plays a crucial role not only in maintaining the quality control of proteins within the ER by chaperoning protein folding but also assumes a vital role in coordinating ER-associated degradation (25). It has been demonstrated that HSP90B1 is closely associated with advanced MM and holds promising prospects as a diagnostic and prognostic marker (26). HSPA5, also known as GRP78, is a member of the heat shock protein 70 (HSP70) family and exhibits similar protein folding chaperone activity. It has been found to be highly expressed in MM and represents a promising therapeutic target in the treatment of this disease (27, 28). However, current research on ER stress and molecular chaperones in the context of drug resistance in MM primarily focuses on classical drugs such as bortezomib and lenalidomide (25, 29). Our study, for the first time, identifies a subgroup characterized by ER stress activation and upregulation of molecular chaperones, which exhibits resistance to CAR-T therapy. This finding suggests the conservation of this resistance mechanism in MM and further highlights its potential as a therapeutic target in MM.

In addition to tumor cell factors, T cell immunity has been highlighted as a crucial factor for the response to CAR-T therapy. CAR-T cells, namely CAR-engineered T cells, not only possess their own recognition and cytotoxic functions but also facilitate the activation and proliferation of endogenous T cells within the host, thereby further augmenting anti-tumor immunity (30, 31). In a study involving one patient with plasma cell leukemia, a highly aggressive plasma cell neoplasm, who received anti-BCMA CAR-T therapy, analysis of scRNA-seq validated the capability of CAR-T cells to recruit endogenous T cells *in vivo*, potentially exerting reciprocal effects on CAR-T cells and modulating the microenvironment (32). However, current research at the single-cell level regarding endogenous T cells after anti-BCMA CAR-T therapy in MM patients remains extremely limited. Given that the dysfunction of T cells contributes to the immunosuppressive immune microenvironment, we analyzed the T cells within the tumor microenvironment and circulating throughout the body during CAR-T therapy. We discovered impaired effector function and enhanced exhaustion in endogenous T cells of relapsed patients, emphasizing the pivotal role of endogenous T cell status in determining the efficacy of CAR-T therapy. Additionally, as essential molecules regulating T cell responses, immune checkpoints play a significant role in balancing immune surveillance of the environment. Co-inhibitory immune checkpoints, such as PD1, CD47, CTLA4 and LAG3, are highly expressed on T cell in a variety type of cancers, leading to attenuated T cell responses and ultimately immune evasion of cancer cells (33). Hence, we conducted a further analysis of immune checkpoint expression in endogenous T cells and found that co-inhibitory immune checkpoints were highly expressed in effector T cells of relapsed patients. Interestingly, we observed a significant biological concordance between peripheral T cells and tumor-infiltrating T cells, suggesting that T cells throughout the body participate in the immune response after CAR-T treatment and influence its efficacy. Interestingly, our analysis of DEGs at various time points during CAR-T therapy revealed that the expression of S100A8 and S100A12 was found to differ across all time points. S100A8 and S100A9 are members of the S100 protein family, characterized by the presence of two canonical EF-hand calcium-binding motifs. These proteins play crucial roles in calcium-dependent regulation, influencing processes such as cell growth, migration, differentiation, and the release of cytokines (34). In a recent study on relapsed or refractory T-cell acute lymphoblastic leukemia/lymphoma patients undergoing anti-CD7 CAR-T therapy, scRNA-seq demonstrated high expression of S100A8 in various T cell subpopulations in the relapsed patients (35). Additionally, the enrichment of these DEGs indicates that these genes are enriched in the ribosome and immune regulation pathways. This suggests that alterations in T cell responses and functions may represent crucial mechanisms influencing the outcomes of CAR-T therapy. Further research is required to confirm the regulatory roles of molecules, including the S100 protein family, in endogenous T-cell regulation following CAR-T therapy.

The intercellular communication between tumor cells and T cells has emerged as a prominent topic of investigation within the realm of CAR-T therapy (30, 36). In this regard, we conducted an analysis of cell-cell interaction between endogenous T cells and tumor cells in patients, employing a ligand-receptor pairing-based approach. Inhibitory signaling pairs, including SIRPG-CD47 and TGFB1-TGFBR3 (37), have been found to be significantly expressed in relapsed patient. This observation aligns with the aforementioned findings of highly expressed checkpoints and impaired functionality in endogenous T cells of relapsed patient, further corroborating the notion that impaired endogenous T cell function can impact the efficacy of anti-BCMA CAR-T therapy. Furthermore, we have also observed variations in CD74-associated ligand-receptor pairs among patients with different treatment responses. CD74 serves as a chaperone in antigen presentation by mediating the assembly and subcellular trafficking of the MHC-II complex, and it is overexpressed in various types of tumors (38). Interestingly, TGFβ has been reported to regulate CD74 in tumors (39, 40). Therefore, the TGFβ/CD74 pathway might represent a novel mechanism influencing the efficacy of anti-BCMA CAR-T therapy and could potentially emerge as a promising therapeutic target.

## 5 Conclusion

In summary, we conducted single-cell RNA sequencing on patients undergoing anti-BCMA CAR-T therapy, depicting the cellular atlas of the immune microenvironment within the bone marrow and peripheral blood. We identified a population of MM cells resistant to anti-BCMA CAR-T therapy and demonstrated that attenuated effector function and elevated expression of immune checkpoint of T cells contribute to the relapse following anti-BCMA CAR-T therapy. However, this study still has certain limitations. On one hand, the sample size is relatively small; and on the other hand, due to the limited availability of separable CAR-T cells within the body three months after infusion, we did not analyze the CAR-T cells themselves. Nevertheless, this study still offers novel insights into the changes in endogenous immune cells, particularly in the relatively long term after CAR-T therapy. Further validation through larger sample sizes and foundational experimental studies is warranted to confirm our findings and provide more avenues for overcoming resistance following anti-BCMA CAR-T therapy.

## Data availability statement

The original contributions presented in the study are included in the article/**Supplementary Materials**, further inquiries can be directed to the corresponding author/s.

## Ethics statement

The manuscript presents research on animals that do not require ethical approval for their study.

## Author contributions

YX: Conceptualization, Data curation, Formal Analysis, Methodology, Resources, Software, Validation, Visualization, Writing – original draft. QZ: Data curation, Software, Writing – original draft. XS: Data curation, Investigation, Writing – original draft. YJ: Resources, Writing – original draft. JW: Data curation, Writing – original draft. JZ: Data curation, Funding acquisition, Writing – original draft. LC: Conceptualization, Funding acquisition, Project administration, Resources, Supervision, Writing – review & editing.
