# Supplementary material for: Single-cell transcriptomic atlas throughout anti-BCMA CAR-T therapy in patients with multiple myeloma
Source: Front Immunol. 2023 Nov 14;14:1278749. doi: 10.3389/fimmu.2023.1278749 (PMC10682082; doi:10.3389/fimmu.2023.1278749)
Supplement: Supplementary file 1 [file DataSheet_1.docx]

Supplementary Material

# Supplementary Tables

**Table S1.** Quality statistics data.

**Table S2.** Signature related gene sets used for clustering.

**Table S3.** Gene sets used for calculating the UCell scores.

**Table S4.** Differentially expressed genes of B cell clusters (TOP 50).

**Table S5.** Differentially expressed genes (DEGs) of CD8^+^Teff between non-relapsed (P1&P2) and relapsed (P3) at different time points in anti-BCMA CAR-T tharepy (pre, 3 months, 6 months and 9 months). **(A)** DEGs at 0m. **(B)** DEGs at 3m. **(C)** DEGs at 6m. **(D)** DEGs at 9m.

**Table S6.** Intersection of differentially expressed genes of CD8^+^Teff in non-relapsed (P1&P2) and relapsed (P3) patients.

# Supplementary Figures


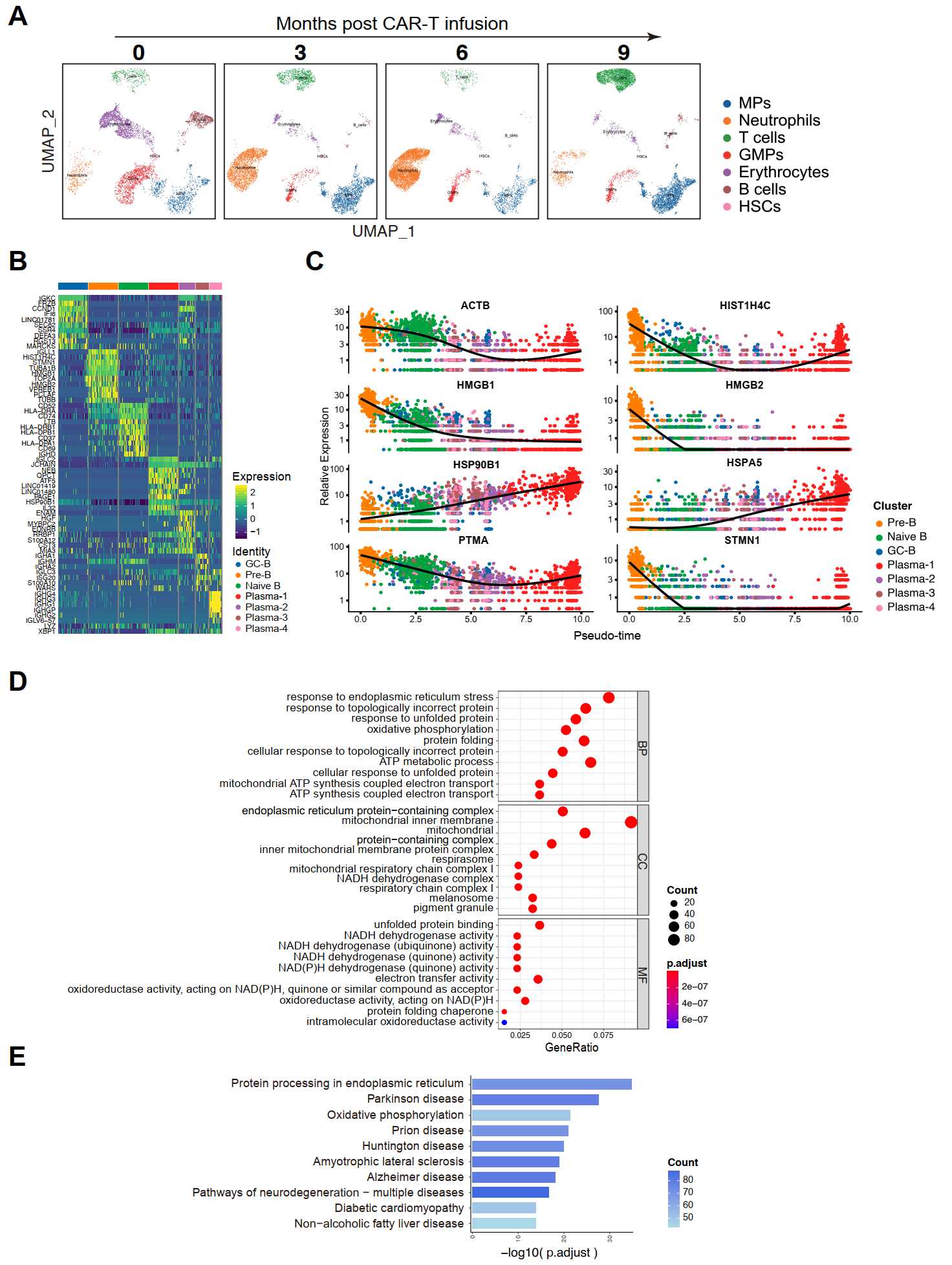


**Figure S1.** Characterizing the anti-BCMA CAR-T therapy resistant tumor cells in the relapsed patient. **(A)** UMAP of bone marrow cells from relapsed patient (P3) based on the time point of specimen collection (pre, 3 months, 6 months and 9 months). **(B)** Heatmap of differentially expressed genes of each identified cluster of the bone marrow B cells. **(C)** Eight genes with the strongest statistical association with pseudotime trajectories. **(D)** Dotplot of GO enrichment for differentially expressed genes in Plasma-1. **(E)** Barplot of KEGG enrichment for differentially expressed genes in Plasma-1.


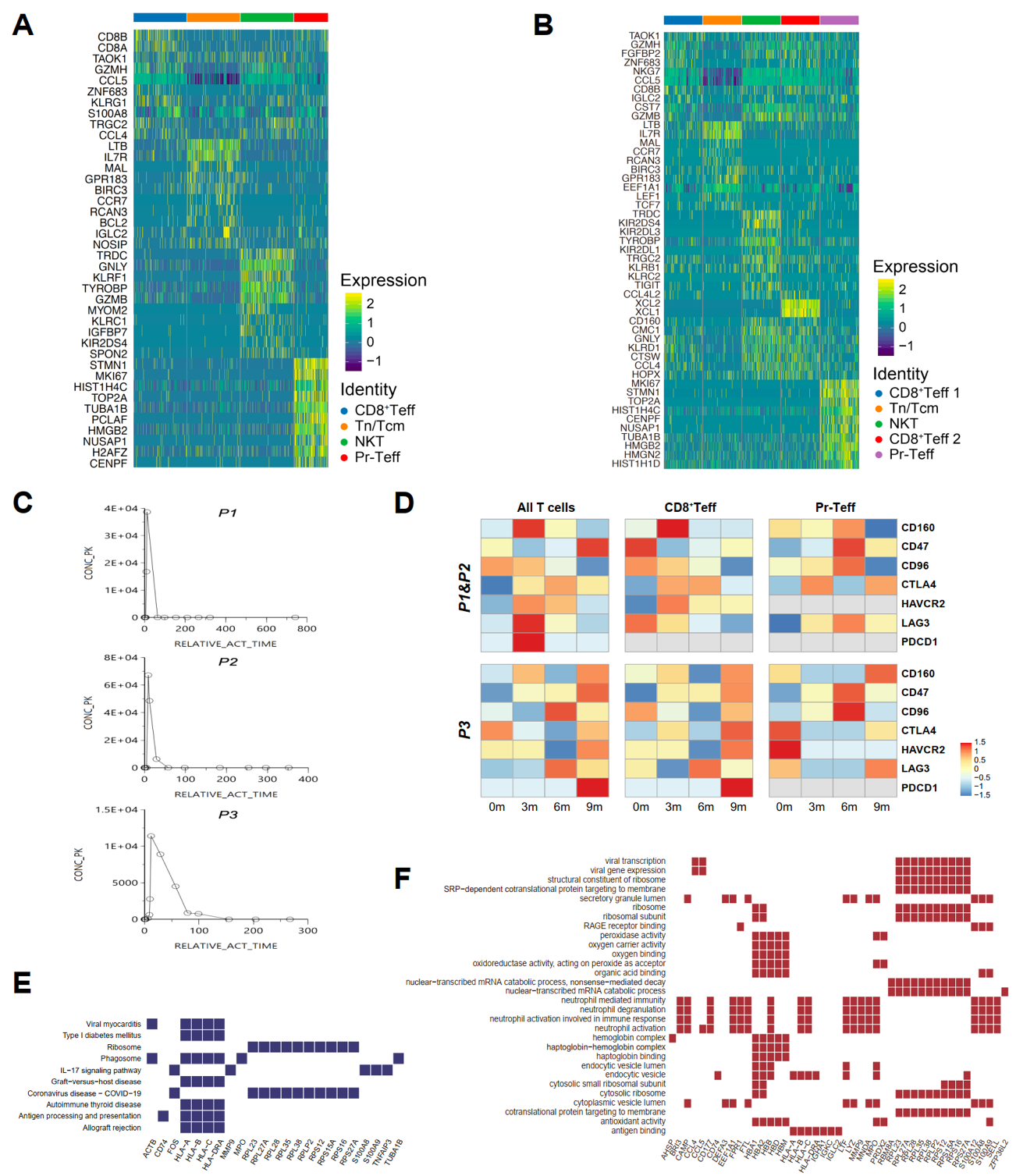


**Figure S2.** Features of T cells in anti-BCMA CAR-T therapy. **(A)** Heatmap of differentially expressed genes of each identified cluster of the bone marrow T cells. **(B)** Heatmap of differentially expressed genes of each identified cluster of the peripheral blood T cells. **(C)** CAR-T cell expansion curve for patients assessed by means of qPCR assay in peripheral blood. The horizontal axis represents the number of days following CAR-T infusion, and the vertical axis represents the quantitative measurement of CAR-T cells (copies/μg of genomic DNA). **(D)** Heatmap of immune checkpoint expression in bone marrow T cells and the effector subclusters along CAR-T therapy. **(E)** Heatplot of enriched KEGG pathways and relevant genes in the enrichment for differentially expressed genes in bone marrow CD8^+^Teff that appear at two or more time points. **(F)** Heatplot of enriched GO terms and relevant genes in the enrichment for differentially expressed genes in bone marrow CD8^+^Teff that appear at two or more time points.


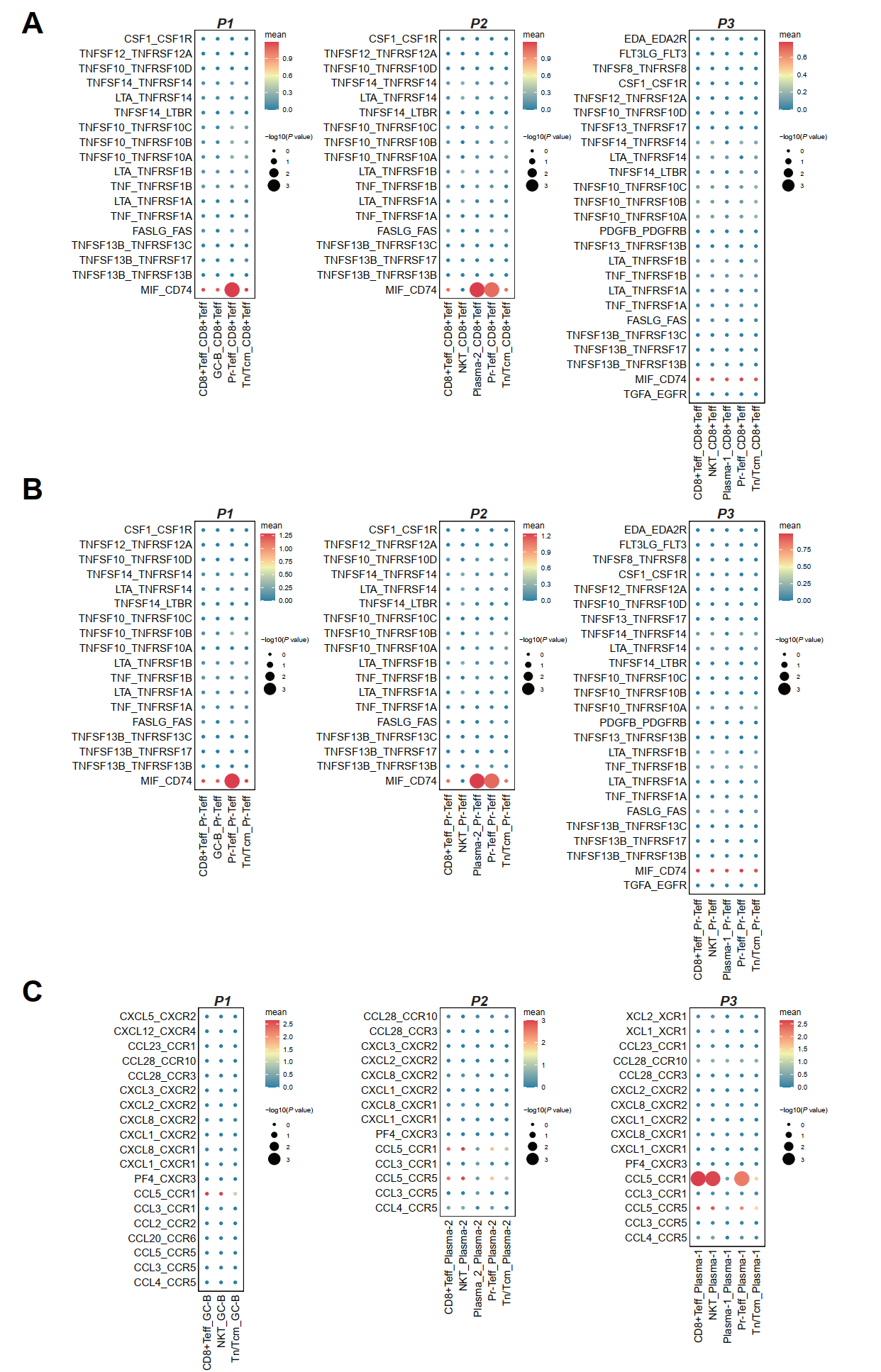
**Figure S3.** Cell communication between bone marrow T cells and tumor cells in anti-BCMA CAR-T therapy regarding cytokines or chemokines. **(A)** Dotplot showing the top ligand-receptor pairs of cytokines based on receptors in CD8^+^Teff. **(B)** Dotplot showing the top ligand-receptor pairs of cytokines based on receptors in Pr-Teff. **(C)** Dotplot showing the top ligand-receptor pairs of chemokines based on receptors in tumor cells.


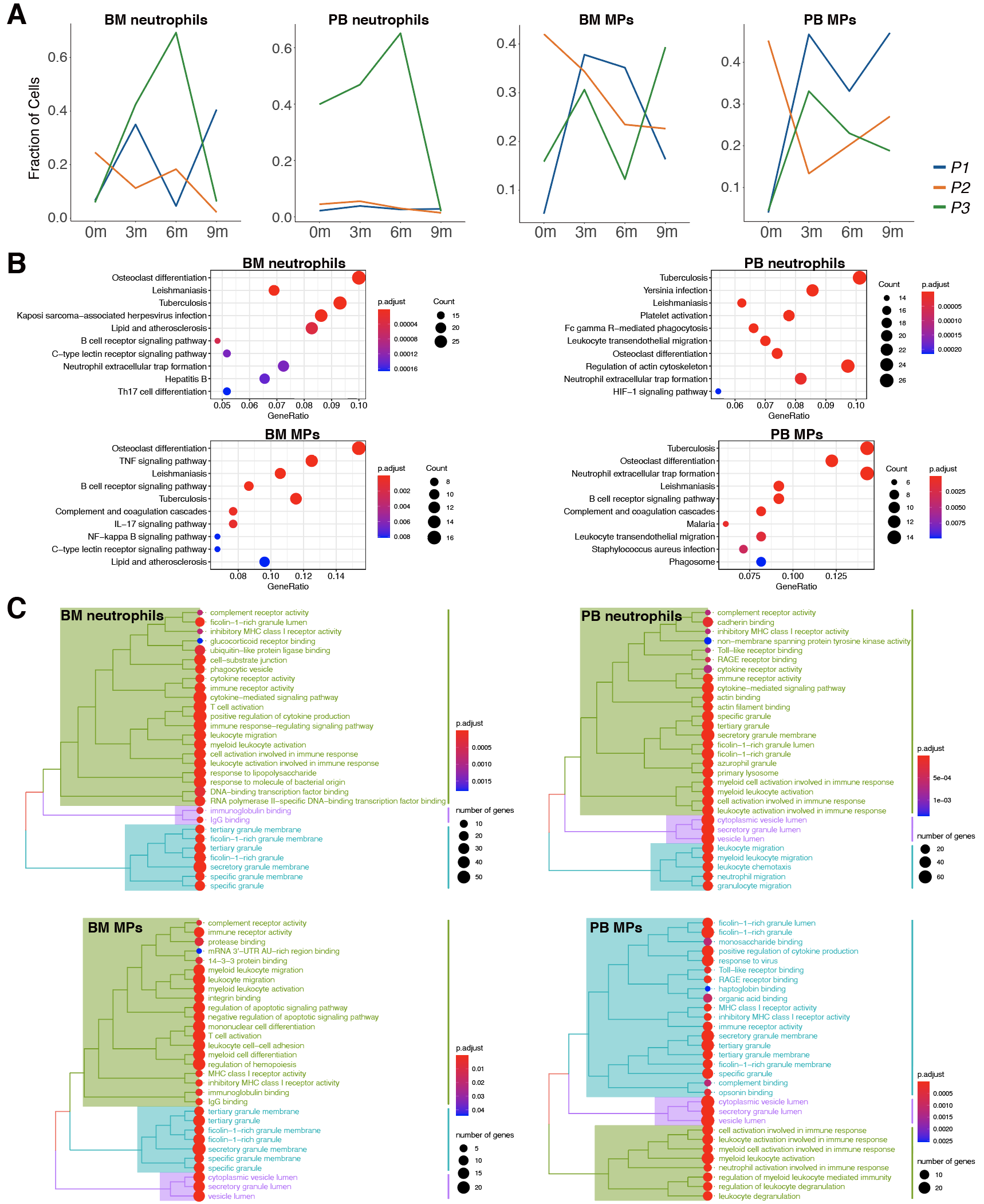


**Figure S4.** Differences in quantity and function of neutrophils and MPs between relapsed and non-relapsed patients. **(A)** Changes in proportions of neutrophils and MPs in the BM and PB over time. **(B)** Dotplot of KEGG enrichment for DEGs between relapsed and non-relapsed patients in each cell clusters. **(C)** Treeplot of GO enrichment for DEGs between relapsed and non-relapsed patients in each cell clusters.


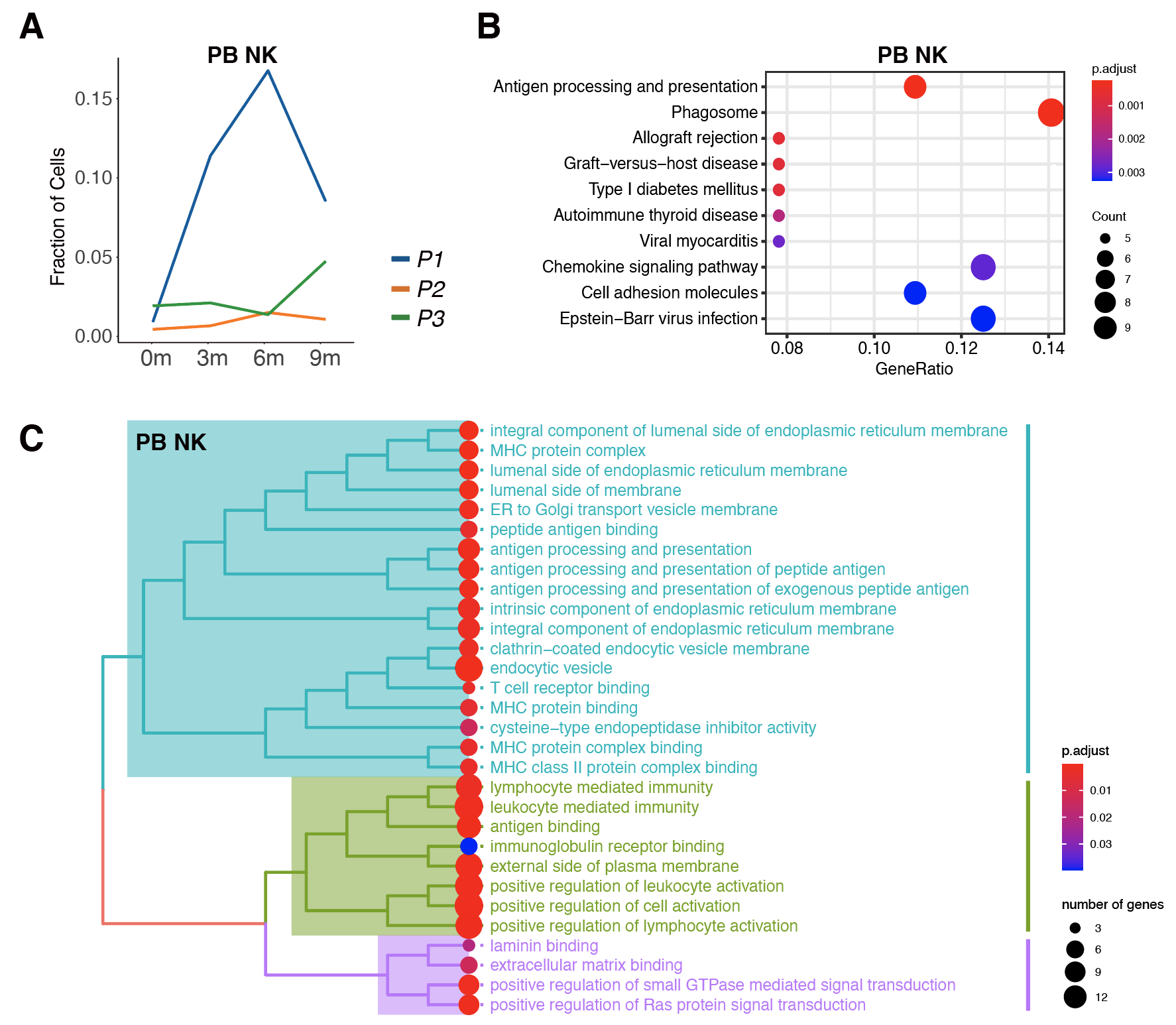


**Figure S5.** Differences in quantity and function of peripheral NK cells between relapsed and non-relapsed patients. **(A)** Changes in proportions of NK cells over time. **(B)** Dotplot of KEGG enrichment for DEGs between relapsed and non-relapsed patients. **(C)** Treeplot of GO enrichment for DEGs between relapsed and non-relapsed patients.
